# Aβ40 Oligomers Identified as a Potential Biomarker for the Diagnosis of Alzheimer's Disease

**DOI:** 10.1371/journal.pone.0015725

**Published:** 2010-12-30

**Authors:** Carol Man Gao, Alice Y. Yam, Xuemei Wang, Erika Magdangal, Cleo Salisbury, David Peretz, Ronald N. Zuckermann, Michael D. Connolly, Oskar Hansson, Lennart Minthon, Henrik Zetterberg, Kaj Blennow, Joseph P. Fedynyshyn, Sophie Allauzen

**Affiliations:** 1 Research and Development, Novartis Vaccines and Diagnostics, Emeryville, California, United States of America; 2 Clinical Neurochemistry Laboratory, Department of Neuroscience and Physiology, Sahlgrenska University Hospital, Mölndal, Sweden; 3 Clinical Memory Research Unit, Department of Clinical Sciences Malmö, Lund University, Malmö, Sweden; Federal University of Rio de Janeiro, Brazil

## Abstract

Alzheimer's Disease (AD) is the most prevalent form of dementia worldwide, yet the development of therapeutics has been hampered by the absence of suitable biomarkers to diagnose the disease in its early stages prior to the formation of amyloid plaques and the occurrence of irreversible neuronal damage. Since oligomeric Aβ species have been implicated in the pathophysiology of AD, we reasoned that they may correlate with the onset of disease. As such, we have developed a novel misfolded protein assay for the detection of soluble oligomers composed of Aβ x-40 and x-42 peptide (hereafter Aβ40 and Aβ42) from cerebrospinal fluid (CSF). Preliminary validation of this assay with 36 clinical samples demonstrated the presence of aggregated Aβ40 in the CSF of AD patients. Together with measurements of total Aβ42, diagnostic sensitivity and specificity greater than 95% and 90%, respectively, were achieved. Although larger sample populations will be needed to confirm this diagnostic sensitivity, our studies demonstrate a sensitive method of detecting circulating Aβ40 oligomers from AD CSF and suggest that these oligomers could be a powerful new biomarker for the early detection of AD.

## Introduction

Alzheimer's Disease (AD) is a neurodegenerative disorder characterized by progressive memory loss and cognitive dysfunction. It is the most prevalent form of dementia, estimated to affect 13 million people worldwide [1]. While the precise mechanism underlying the disease is not fully understood, the aggregation of amyloid beta (Aβ) appears to play an important role [2]–[4]. Aβ peptides of various lengths (typically 1–40 and 1–42) are cleavage products of the amyloid precursor protein that aggregate and form insoluble plaques in AD brains. Post mortem identification of these plaques together with neurofibrillary tangles and neuronal loss is currently the definitive and only fully accepted diagnostic confirmation of AD [5], [6]. However, recent reports suggest that smaller, soluble Aβ oligomers are more likely to be the pathogenic agents of disease [3], [4], [7]–[10].

A growing number of in vitro generated oligomers of varied size and structure have been implicated in AD [4]. However, the actual identity of the oligomer participating in AD pathogenesis remains elusive. Its chemical composition is also poorly defined, although several lines of evidence suggest that AD-associated oligomers are primarily composed of Aβ42 [3]. For instance, one unifying feature of AD is the presence of Aβ42-containing plaques in the brain parenchyma [11], [12]. This suggests that any soluble oligomers would also be composed of Aβ42. In addition, Aβ42 appears to be more amyloidogenic than Aβ40 and is found more frequently in plaques despite existing at much lower physiological concentrations [13]. Lastly, several presenilin mutations linked to familial forms of AD are known to increase production of Aβ42 cleavage products [14], further implicating this Aβ peptide in pathogenesis. Consequently, it is generally assumed that cytotoxic oligomers mediating AD are composed of Aβ42 peptides.

While the precise conformation of in vivo oligomers is unknown, several lines of evidence suggest that aggregated proteins share a number of structural properties. For instance, amyloid fibrils composed of different proteins have similar cross beta sheet structure, allowing binding and detection by a number of compounds such as Thioflavin T and Congo Red [15], [16]. Smaller aggregated species such as oligomers and protofibrils also share structural properties recognized by conformation-sensitive antibodies [17], [18]. Similarly, we have recently developed a series of peptides for the selective capture of aggregated prion protein (PrP) from plasma [19]. We now report the adaptation and integration of these peptides into a Misfolded Protein Assay (MPA) and the capture of aggregated Aβ from CSF. Given that soluble Aβ oligomers have been reported in the CSF of AD patients [10], [20]–[22], we reasoned that the MPA could be utilized to detect oligomers found in vivo. Using this technology, we demonstrate for the first time the presence of Aβ40 oligomers in AD CSF. We propose that Aβ40 oligomers could be a novel biomarker for the early diagnosis of AD.

## Methods

### MPA capture of Aβ aggregates

**Figure 1:**
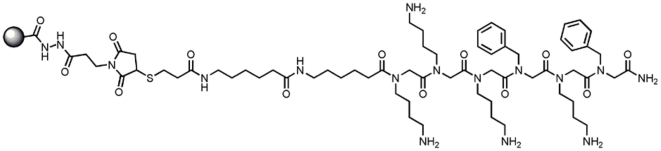
Aggregate Specific Reagent 1 (ASR1). Chemical structure of ASR1 peptoid conjugated to a solid surface.

Aggregate Specific Reagent 1 (ASR1) beads were generated by chemical conjugation of a thiolated ASR1 derivative to Dynal M270 carboxyl beads (Invitrogen Dynal AS, Oslo, Norway, 30 mg/mL) via maleimide chemistry (Figure 1). Control beads consisted of similarly conjugated glutathione molecules.

**Figure 2:**
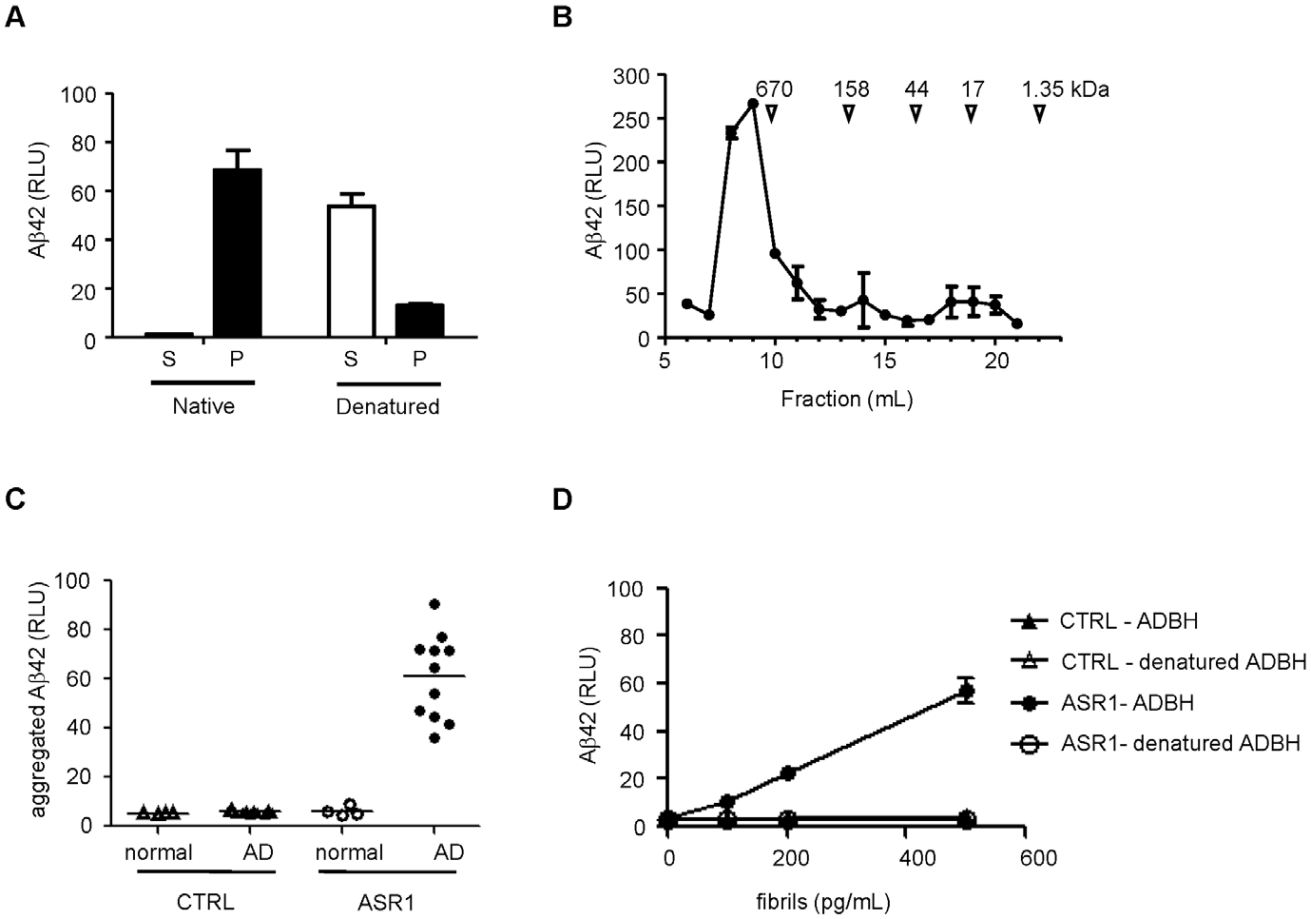
MPA detection of a conformational epitope in Aβ aggregates from AD brain homogenates (ADBH). *A.* ADBH was centrifuged at 134,000*g* for 1 hr, and denatured Aβ42 in the supernatant (S) and pellet (P) fractions was detected by ELISA. *B.* ADBH was fractionated by size exclusion chromatography and Aβ42 was detected by ELISA. *C.* 75 nl of normal (open symbols) or AD (closed symbols) brain homogenate was subjected to the MPA using control (triangles) or ASR1-coated (circles) beads. *D.* Aβ aggregates from an ADBH were examined by the MPA with (open symbols) or without (filled symbols) a pretreatment with 5.4 M guanidine thiocyanate using control (triangles) or ASR1-coated (circles) beads. Error bars represent the standard deviation of triplicate reactions.

Aβ42 aggregates from AD brain homogenate (ADBH) were captured with 3 µl ASR1 beads for 1 hour at 37°C in 100 µl 80% plasma by spiking with 75 nl of 10% brain homogenate (Fig. 2C) or the indicated concentrations of Aβ aggregates (Fig. 2D) in capture buffer (50 mM Tris, 150 mM NaCl, 1% Tween-20, 1% Triton X-100 pH 7.5). 10% ADBH was estimated to contain ∼1 pg/nL Aβ42 aggregates (data not shown). The beads were then washed with TBST (50 mM Tris, 150 mM NaCl, 0.05% Tween-20 pH 7.5), and bound proteins were eluted with 0.1 M NaOH at 80°C for 30 minutes and neutralized with 0.12 M NaH_2_PO_4_, 0.4% Tween-20. The eluted Aβ was subsequently detected by an Aβ42-specific ELISA, utilizing an Aβ x-42 specific capture antibody (12F4, Covance, Princeton, NJ) and HRP-conjugated 4G8 antibody (Covance) for detection. To test the conformational nature of ASR1 binding, ADBH was pretreated with 5.4 M guanidine thiocyanate for 30 minutes at room temperature prior to dilution into 80% plasma and MPA detection. AD and control brain samples were obtained from the tissue bank of the Swiss National Reference Centre of Prion Diseases (Zürich, Switzerland).

In vitro Aβ42 oligomers spiked into normal CSF and in vivo oligomers from clinical AD CSF were similarly detected by the MPA with a few modifications. 250 µl 80% human CSF in capture buffer was incubated with 30 µl ASR1 beads. ASR1 beads were subsequently washed with TBST and 1% Zwittergent 3-14 before Aβ42 and Aβ40 were eluted and detected in a multiplex format by MSD immunoassay (Meso Scale Discovery, Gaithersburg, Maryland) according to the manufacturer's instructions. In vitro Aβ42 oligomers are estimated to be 12mers and were prepared as described [23]. Normal pooled CSF for oligomer spiking experiments was purchased from Analytical Biological Services Inc. (Wilmington, DE).

### Biochemical characterization of brain-derived aggregates

Solubility of brain-derived Aβ aggregates was assessed by differential centrifugation. 250 nl 10% ADBH diluted into 0.5x capture buffer was treated with or without 3 M guanidine thiocyanate for 10 minutes at 95°C and then centrifuged for 1.5 hour at 134,000 *g* at 4°C. Aβ42 in supernatant and pellet fractions were denatured with 3 M guanidine, diluted with TBST buffer, and detected by ELISA.

The size of brain-derived Aβ42 aggregates was estimated by separation of 75 µl of 10% ADBH on a Superdex200 10/300 GL column at a flow rate of 1 mL/min in TBS with 1 mM EDTA, 1% NP40. 1 mL fractions were collected and denatured with 0.1 N NaOH as stated above prior to ELISA detection.

### ELISA

ELISA plates were coated with 2 µg/mL 12F4 antibody in coating buffer (0.1 M NaH_2_PO_4_, 1% NaCl, pH 6), washed with TBST, and blocked with 1% BSA, 3% sucrose in TBS for 1 hour at 37°C. 0.2 µg/mL HRP-conjugated 4G8 antibody in conjugate diluent (0.1% BSA, 0.01% casein in PBS) was diluted 1∶1 with the sample and applied to the 12F4-coated plates for 1 hour at room temperature before detection by a chemiluminescent substrate (Pierce Supersignal ELISA Femto Substrate, Thermo Fisher Scientific, Rockford, IL).

### Patients and CSF sampling

The AD group consisted of 26 patients consulting the memory disorder clinic at Malmö University Hospital, Sweden, mean age 71.8±7.3 years. All patients underwent physical, neurological and psychiatric examination, cognitive tests, careful clinical history and functional assessment. Patients diagnosed with AD had to meet the DSM-IIIR criteria of dementia [24] and the criteria of probable AD defined by NINCDS-ADRDA [25]. The AD patients were followed over time with repeated clinical evaluations, which increases the clinical diagnostic accuracy. The control group, in total 10 cases, mean age 69.4±9.7 years, was defined based on absence of memory complaints or any other cognitive symptoms, and no signs of active neurological or psychiatric disease. All patients and controls gave informed consent to participate in the study. The study was conducted according to the provisions of the Helsinki Declaration and was approved by the ethics committee of Lund University, Sweden. CSF was collected in polypropylene tubes, centrifuged, aliquoted, and stored at −80°C pending analyses.

### Statistical analysis

Statistical comparison of two populations was performed using two-tailed t-test using GraphPad Prism for Windows, v 5.01 (GraphPad Software, San Diego, CA). Receiver operating characteristic curves (ROC) were generated using R (R Foundation for Statistical Computing, Vienna, Austria).

## Results

### An aggregate-specific reagent captures Aβ aggregates from AD brain homogenate in a conformation-dependent manner

We have developed an Aggregate Specific Reagent (ASR1, Figure 1) that preferentially binds aggregated proteins over monomeric proteins. ASR1 is a peptoid, a peptidomimetic containing N-substituted glycines [26], which have been shown to be resistant to proteolytic digestion from enzymes commonly found in body fluids [27]. The ASR1 sequence is derived from a PrP peptide that has a strong ability to capture aggregated PrP in solution [19]. ASR1 can capture picomolar amounts of insoluble aggregated PrP from a solution containing an excess of normally folded PrP (Figure S1 and [19]).

As amyloid aggregates are characterized by similar cross-beta sheet structure, they share conformational epitopes that are recognized by amyloid-binding molecules such as Congo Red and thioflavin T [15], [16] as well as conformation-specific antibodies [17], [18]. We hypothesized that ASR1 might also recognize structural epitopes common to aggregated proteins rather than epitopes specific to PrP aggregates. As such, we asked if ASR1 could bind aggregates composed of Aβ. Like brain-derived PrP aggregates, aggregates from an AD brain homogenate (ADBH) were insoluble at 134,000 *g* (Figure 2A), suggesting that they were large. Fractionation by size exclusion chromatography demonstrated that these aggregates were larger than a 670 kDa molecular weight standard (Figure 2B).

To test if ASR1 could bind to Aβ aggregates, ASR1 beads were incubated with AD and normal brain homogenates, and then the bound Aβ42 was eluted, denatured, and then detected by an Aβ42-specific ELISA. This method, the MPA, could clearly distinguish between control and AD samples, while a parallel assay using a control bead could not (Figure 2C). Furthermore, ASR1 capture of Aβ42 was conformation-dependent since treatment of the aggregates with a chemical denaturant prior to the MPA abolished all binding (Figure 2D).

### ASR1 captures soluble oligomers in CSF

**Figure 3:**
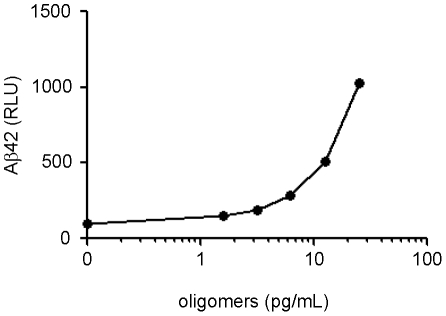
MPA detection of synthetic oligomers at subpicomolar levels. In vitro synthesized Aβ42 oligomers were diluted into normal CSF at the indicated concentrations and detected by the MPA. Error bars represent the standard deviation of triplicate reactions.

We reasoned that ASR1 would be a powerful tool for early diagnosis of AD if, in addition to the large aggregates found in AD brain tissue, it could also capture the smaller oligomers implicated in AD pathogenesis. As such, we tested whether ASR1 could capture Aβ oligomers diluted into CSF. In vitro oligomers composed of ∼12 Aβ42 monomers were prepared as described [23] and diluted at varying concentrations into normal CSF before being subjected to the MPA (Figure 3). Of note, the MPA could detect the oligomers with high sensitivity with a limit of detection of 5 pg/mL Aβ42 (corresponding to ∼80 fM oligomer).

### ASR1 detects Aβ40 oligomers in AD CSF

**Figure 4:**
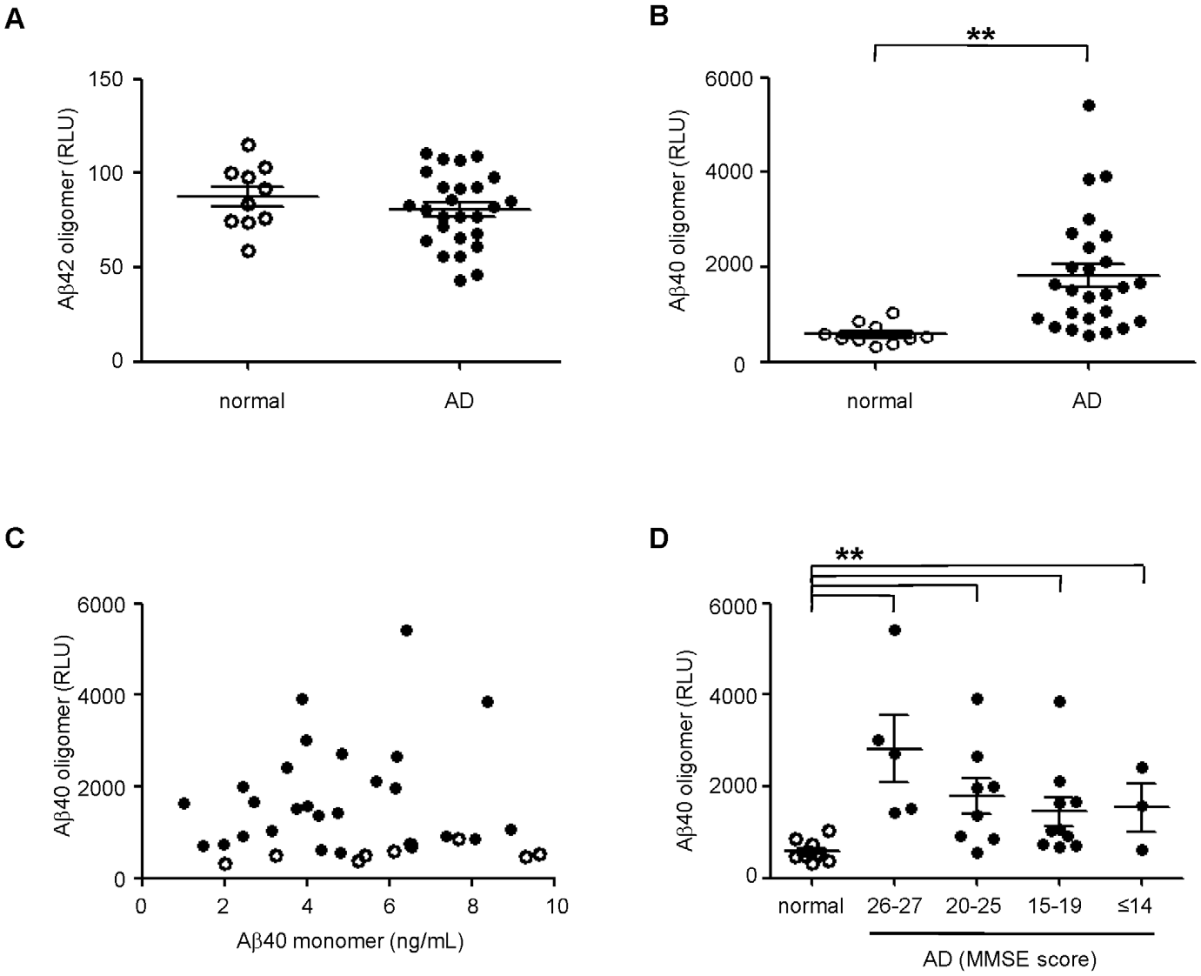
MPA detection of Aβ40 oligomers from AD CSF. Normal (open circles) and AD (closed circles) CSF were analyzed by the MPA. Oligomers containing Aβ42 *(A)* and Aβ40 *(B)* were captured by ASR1 beads followed by detection using a multiplex immunoassay. Significant differences between normal and AD population were calculated by t-test (Aβ42, *p* = 0.31; Aβ40, **p<0.01). *C.* Lack of correlation between MPA-detected Aβ40 signal and the concentration of Aβ40 monomers in AD CSF (R^2^ = 0.037). *D.* The distribution of Aβ40 oligomers by disease severity was examined by categorization of AD patients according to MMSE scores (**p<0.01 between normal and all AD groups). The mean and standard error of the mean (SEM) are shown for all groups.

Because we achieved significant sensitivity at detecting in vitro oligomers spiked into normal CSF, we asked whether the MPA could detect endogenous oligomers reported to be present in AD CSF [20]–[22]. CSF from 26 clinically diagnosed AD patients at varying stages of disease and 10 aged-matched controls were examined by the MPA. As before, CSF was incubated with ASR1 beads and the captured Aβ was detected by a multiplex immunoassay specific for Aβ40 and Aβ42. Upon examination of captured Aβ42, we did not detect any difference between AD and control populations (Figure 4A). However, we observed surprisingly clear and significant differences in the Aβ40 signal between the two groups (Figure 4B). Importantly, Aβ40 signals from the MPA did not correlate with the concentration of total Aβ40 in the CSF (presumably this immunoassay format would bias the detection of total Aβ toward monomeric species, so they are indicated as such henceforth). This suggests that the Aβ40 signals were associated with specific MPA oligomeric capture and not nonspecific binding of Aβ40 monomers to the capture beads (Figure 4C). When we further categorized the AD samples into groups of increasing disease severity based on clinical Mini-Mental State Examination (MMSE) scores, we found that the Aβ40 oligomers were not only found in individuals with late-stage AD and low MMSE scores but also in patients with early stage AD and higher MMSE scores (Figure 4D). Therefore, using the MPA, we have identified Aβ40 oligomers as a potential biomarker that could diagnose AD in the early stages of disease.

### Aβ40 oligomers are a novel biomarker for the diagnosis of AD

**Figure 5:**
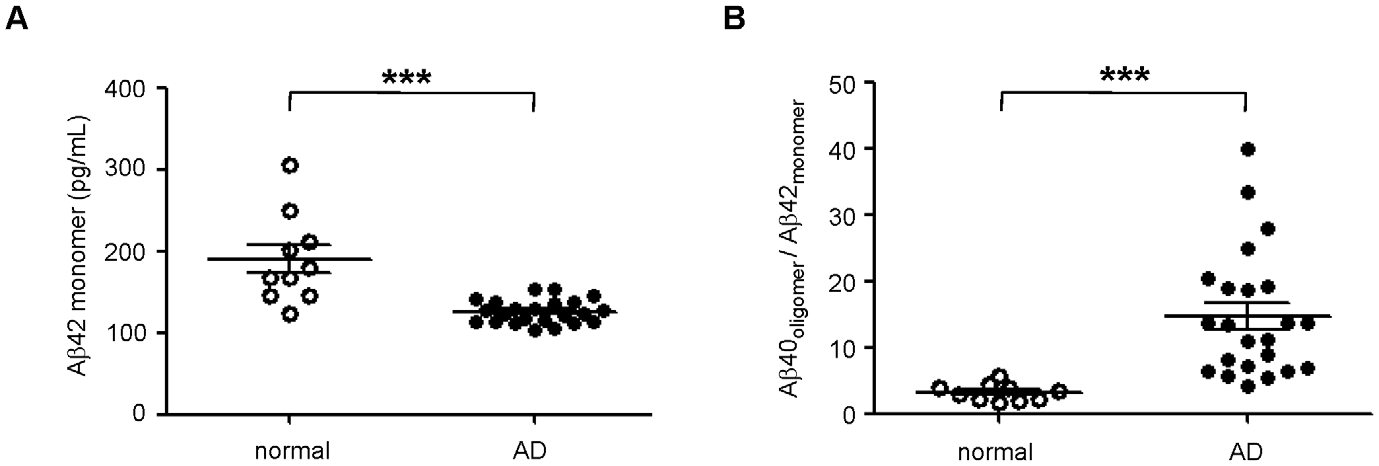
Synergistic combination of Aβ40 oligomer signal with monomeric Aβ42 concentration. *A.* The concentration of monomeric Aβ42 was measured in normal (open circles) and AD (closed circles) CSF (***p<0.001). *B.* The ratio of Aβ40 oligomers (RLU) and Aβ42 concentration (pg/mL) was calculated and plotted for both normal (open circles) and AD (closed circles) populations (***p<0.001). Three AD samples were not included in this analysis because of insufficient sample to measure Aβ42 concentrations. The mean and SEM are shown for all groups.

**Figure 6:**
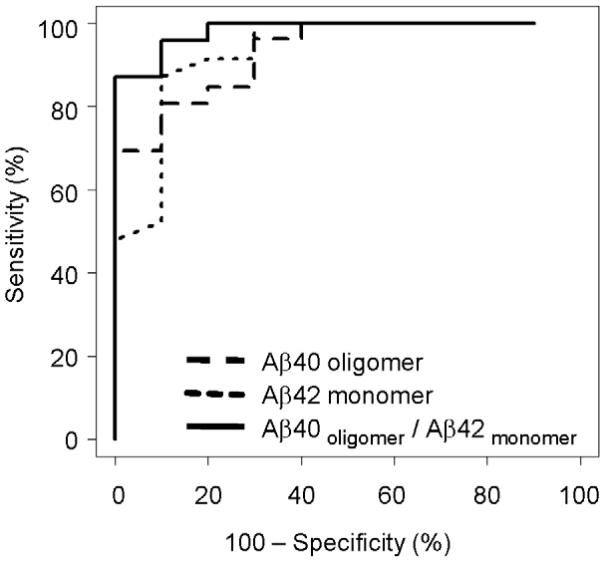
Receiver operating characteristic (ROC) curve. ROC analysis was performed to compare the diagnostic value of 3 biomarkers: oligomeric Aβ40 (– –), monomeric Aβ42 (- - -), and the ratio Aβ40_oligomer_/Aβ42_monomer_ (––).

To validate our results against other investigated biomarkers for AD, we also measured the levels of monomeric Aβ42 in this set of samples. As expected, Aβ42 was significantly decreased in the CSF of AD patients relative to normal individuals (Figure 5A), consistent with previously published results [28]. Since low levels of monomeric Aβ42 and high levels of oligomeric Aβ40 are linked to AD, we combined the two biomarkers to strengthen their predictive value. The resulting ratio of oligomeric Aβ40 to monomeric Aβ42 indeed enhanced the differentiation of control and AD populations (Figure 5B). Additional ROC analysis demonstrated diagnostic sensitivity and specificity of 95% and 90%, respectively (Figure 6, Table 1). Positive and negative predictive value was estimated to be near 95% and 90%, respectively, whereas oligomeric Aβ40 and monomeric Aβ42 biomarkers alone had negative predictive values that were equal to or less than 75% (Table 1).

**Table 1:** ROC analysis and diagnostic performance for Aβ40 oligomer, Aβ42 monomer, and Aβ40_oligomer_/Aβ42_monomer_ biomarkers.

Parameters	Aβ40 oligomer	Aβ42 monomer	Aβ40_oligomer_/Aβ42_monomer_
**ROC AUC**	0.93	0.93	0.98
**Threshold value**	850 RLU	150 pg/ml	4.3
**Sensitivity (%)**	81	87	96
**Specificity (%)**	90	90	90
**Test accuracy (%)**	83	88	94
**Positive predictive value (%)**	95	95	96
**Negative predictive value (%)**	64	75	90

## Discussion

AD is a growing epidemic that impacts nearly 50% of our elderly population greater than 85 years old [29]. However, the development of therapeutics to treat the disease has been hampered by the absence of suitable biomarkers to diagnose the disease. Currently, a clinical diagnosis of AD can only be confirmed with the identification of Aβ plaques in postmortem brain tissue [5], [6]. Premortem clinical diagnosis is also problematic as it relies heavily on subjective reporting and observed cognitive decline that can occur over a period of years. Since the first clinical signs of cognitive dysfunction may appear after significant neuronal loss has occurred, there is a compelling need for biomarkers that can diagnose early AD.

Because Aβ oligomers are suggested to play a key role in AD pathogenesis [3], [4], [7]–[10] and because they have been detected in diagnostically relevant body fluids [10], [20]–[22], we reasoned that Aβ oligomers could be a powerful AD biomarker predicting disease progression. However, the concentration of oligomers in CSF remains poorly defined and only a few studies estimate that they may exist at very low levels [21], [22]. Furthermore, these studies did not distinguish between Aβ40 and Aβ42-containing oligomers since they utilized either antibodies recognizing oligomeric structure or oligomer-specific ELISAs that employed capture and detection antibodies recognizing the same amino-terminal epitopes. In this study, we report the development of a novel assay that specifically detected low concentrations of aggregated Aβ in a small population of AD CSF. Because AD is largely correlated with the accumulation of Aβ42 in the brain, we expected to identify oligomers composed of Aβ42. To our surprise, we found only an enrichment of Aβ40-containing oligomers in AD CSF. These oligomers were observed at multiple stages of AD, suggesting that they could be a biomarker for early diagnosis of AD.

Because Aβ42 oligomers are widely implicated in AD pathology, the correlation we observed of AD with Aβ40 but not Aβ42 oligomers was surprising. One explanation is that we detected oligomers associated with the vascular Aβ40 deposits that are seen in patients with cerebral amyloid angiopathy (CAA). CAA is a pathological feature of AD characterized by the cerebrovascular deposition of Aβ peptides with the major species being Aβ40 [30], [31] and has been estimated to impact greater than 90% of AD cases [32], [33]. Therefore, our findings might reflect an increase of oligomeric Aβ40 associated with these Aβ deposits.

Another possibility is that Aβ40 oligomers may be more soluble and reach CSF more readily than Aβ42 oligomers which may rapidly aggregate and deposit in the brain parenchyma. Consequently, high levels of Aβ40 oligomers in the CSF may be a surrogate marker of an amyloidogenic cascade in the brain.

A third explanation for our inability to identify Aβ42 oligomers may be the sensitivity of our detection system. While both Aβ40 and Aβ42 containing oligomers may be present in CSF and captured by ASR1 beads, the subsequent detection of the constituent monomers may be limited by the sensitivity of the immunoassay (Mesoscale detection limits for Aβ40 and Aβ42 are 5 and 8 pg/mL, respectively). Furthermore, the concentration of Aβ42 in CSF is approximately 10-fold lower than that of Aβ40, suggesting that any Aβ42-containing oligomers may exist at concentrations below our detection limit.

Although Aβ40 aggregates have been documented in AD, the biological relevance of Aβ40 oligomers in AD pathogenesis remains unclear. While nearly all cases of AD report Aβ42-containing plaques in the brain, approximately two thirds of them also report Aβ40 deposits [11], and significant levels of Aβ40 have also been found in AD cortical brain tissue [34]. One interpretation of these results is that aggregation of the less soluble Aβ42 would precede and recruit subsequent aggregation of Aβ40, as has been previously suggested [12], [35]. In support of this idea, purification of oligomers from AD brain tissues have isolated both Aβ40, Aβ42, and Aβ40/42 heterodimers [8], [36]. The impact of Aβ40 oligomers on AD pathogenesis is unclear although some in vitro models suggest that Aβ42 oligomers are significantly more cytotoxic [37]. Nevertheless, preparations of soluble Aβ40 are sufficiently toxic to impact long term potentiation in cell culture systems and cognitive function when injected into mice [38].

Additional studies are clearly needed to understand the role of Aβ40 oligomers in AD pathogenesis. Since oligomeric Aβ is suggested to be a direct causative agent of AD, we believe they may be an ideal predictor of disease progression. Indeed, although the results do not reach significance, our data suggest that there could be an inverse correlation between oligomer concentration and disease severity (Figure 4D) similar to the decline that is observed in the Aβ42 levels of AD patients. Future studies will include larger patient populations and prospective CSF sampling to screen for incipient AD. Additionally, if Aβ oligomers are to become a relevant biomarker for AD, its predictive value must be measured in patients with mild cognitive impairment. Nevertheless, our results showing the detection of Aβ40 oligomers in CSF from patients in the initial stages of AD suggest that the MPA could be a sensitive assay for the early diagnosis of AD.

## Supporting Information

Figure S1 **Prion Protein (PrP) captured from plasma spiked with vCJD or normal brain homogenates.** vCJD (closed circle) and normal (open circle) brain homogenates were spiked into normal human plasma at the indicated concentrations and subjected to the MPA. Captured prion protein was eluted and detected by a prion‐specific ELISA. Materials and Methods: vCJD and normal 10% brain homogenates (w/v) (“Blue” and “Clear” samples, respectively, from National Institute for Biological Standards and Control, United Kingdom) were spiked into normal human plasma (SeraCare Life Sciences, West Bridgewater, MA), after which 200 µl of the solution was incubated with 50 µl of 5x capture buffer and 9 µl ASR1 beads for 1 hour at 37°C. The beads were washed and captured prion protein was subsequently eluted, denatured, and detected by sandwich ELISA [19]. The vCJD brain homogenate had an estimated 4 µg/mL of aggregated PrP.
(TIF)
