# Supplementary figures and images for: Aβ40 Oligomers Identified as a Potential Biomarker for the Diagnosis of Alzheimer's Disease
Source: PLoS One. 2010 Dec 30;5(12):e15725. doi: 10.1371/journal.pone.0015725 (PMC3012719; doi:10.1371/journal.pone.0015725)

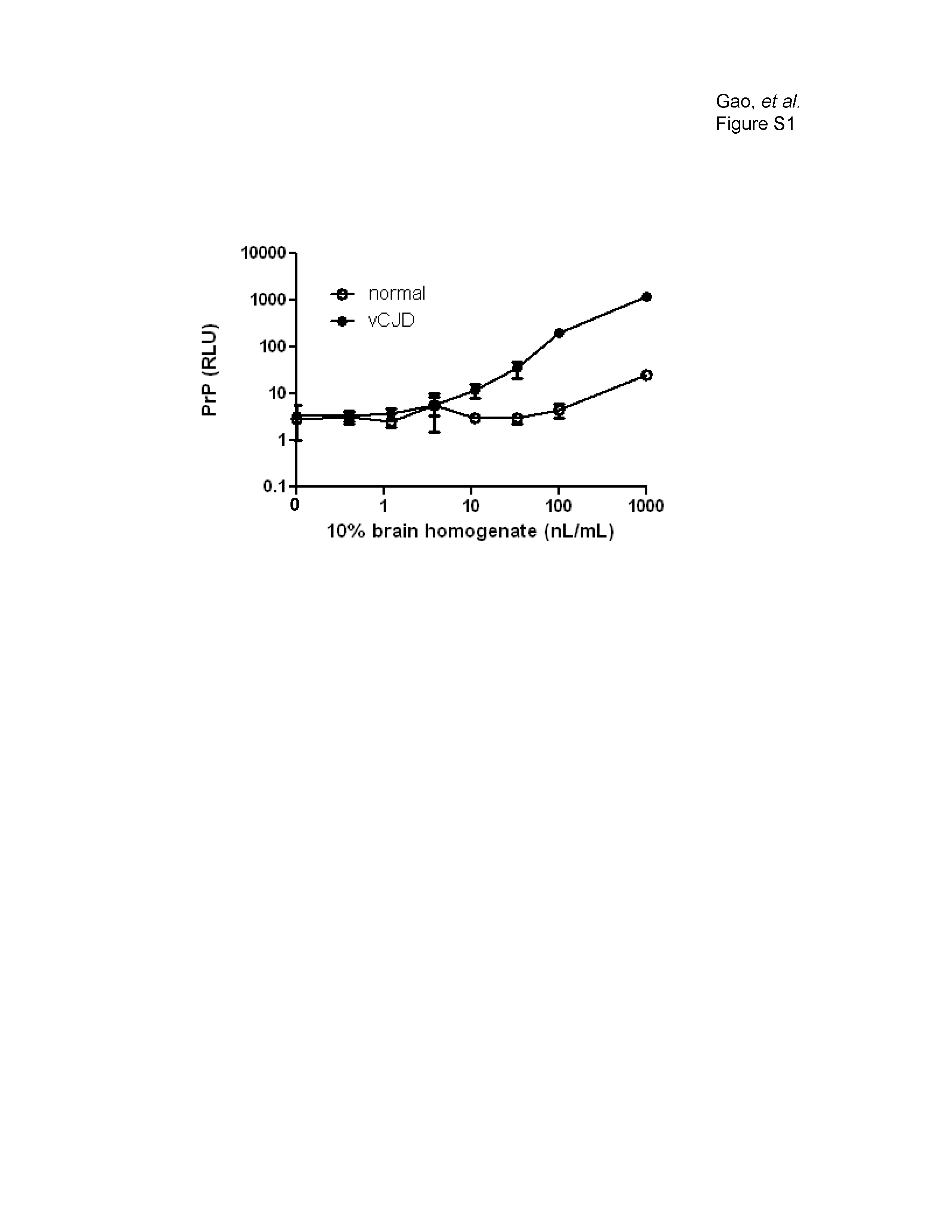

Supplement: Figure S1 — Prion Protein (PrP) captured from plasma spiked with vCJD or normal brain homogenates. vCJD (closed circle) and normal (open circle) brain homogenates were spiked into normal human plasma at the indicated concentrations and subjected to the MPA. Captured prion protein was eluted and detected by a prion‐specific ELISA. Materials and Methods: vCJD and normal 10% brain homogenates (w/v) (“Blue” and “Clear” samples, respectively, from National Institute for Biological Standards and Control, United Kingdom) were spiked into normal human plasma (SeraCare Life Sciences, West Bridgewater, MA), after which 200 µl of the solution was incubated with 50 µl of 5x capture buffer and 9 µl ASR1 beads for 1 hour at 37°C. The beads were washed and captured prion protein was subsequently eluted, denatured, and detected by sandwich ELISA [19]. The vCJD brain homogenate had an estimated 4 µg/mL of aggregated PrP. (TIF) [file pone.0015725.s001.tif]
